# RS-WaterQuality Mapper: an open-source water quality remote sensing toolbox in QGIS

**DOI:** 10.1007/s12145-026-02090-1

**Published:** 2026-03-17

**Authors:** Haibin Su, Hongxing Liu, Lei Wang, Ekaterina Miliutina, Jilin Men, Dan Tian, Yuehan Lu, Song Shu, Richard Beck, Amanjit Premsagar

**Affiliations:** 1 Department of Physics & Geosciences, Texas A&M University, Kingsville, Kingsville, TX 78363 USA; 2 Department of Geography and the Environment, the University of Alabama, Tuscaloosa, AL 35487 USA; 3 Department of Geography and Anthropology, Louisiana State University, Baton Rouge, LA 70803 USA; 4 Department of Geological Sciences, the University of Alabama, Tuscaloosa, AL 35487 USA; 5 Department of Geography and Planning, Appalachian State University, Boone, NC 28608 USA; 6 Department of Geography and Geographic Information Science, University of Cincinnati, Cincinnati, OH 45221 USA

**Keywords:** Water quality, QGIS, Remote sensing, Machine learning, Ensemble model, ACOLITE

## Abstract

Effective water management requires frequent monitoring, but traditional methods are limited in spatiotemporal scope. While satellite remote sensing provides extensive data, a gap persists in accessible, integrated software for non-specialists. To address this, we developed the RS-WaterQuality Mapper, an open-source Python plugin for QGIS. This toolbox provides a complete, scientifically robust workflow for aquatic remote sensing, from aqua-focused atmospheric correction to the application of advanced machine learning models. A key innovation is the implementation of a multi-predictor ensemble model based on spectral-space partitioning, which enhances predictive accuracy in optically complex inland waters. Built on optimized Python libraries and a multi-processing architecture, the tool ensures computational efficiency for processing large satellite scenes. The toolbox’s utility was validated in diverse case studies—a U.S. reservoir, a Kenyan saline lake, and a U.S. river system—demonstrating strong performance (R ^2^>0.80). By embedding state-of-the-art science into a familiar GIS environment, the RS-WaterQuality Mapper empowers a global community of researchers and water resource managers to leverage satellite data for more effective ecosystem management.

## Introduction

Monitoring water quality across inland and coastal systems is essential for protecting ecosystem health, supporting water resource management, and informing policy decisions (Vörösmarty et al. 2010; UNEP 2016). Conventional field-based sampling and laboratory analyses, while providing high analytical precision, are constrained by limited spatial and temporal coverage and substantial logistical costs (Carpenter et al. 2011). Over the past two decades, satellite-based remote sensing has emerged as an indispensable complement, capable of providing synoptic, repeatable, and cost‑effective measurements of key parameters such as chlorophyll‑a, suspended particulate matter, colored dissolved organic matter (CDOM), and turbidity (Palmer et al. 2015; Dörnhöfer and Oppelt 2016; Topp et al. 2020). With the proliferation of moderate‑resolution sensors such as Sentinel-2 MSI (Toming et al. 2016), Landsat-8/9 OLI (Pahlevan et al. 2020), and increasingly frequent commercial constellations such as PlanetScope (Cooley et al. 2017), as well as freely available processing frameworks, water quality mapping has become increasingly feasible.

Despite this progress, operational implementation of remote‑sensing‑based water‑quality assessment remains uneven. Many studies focus on algorithm development or site‑specific validation, while the end‑to‑end integration required for routine monitoring is rarely achieved. Commercial remote sensing image processing packages, such as ENVI or ERDAS Imagine, do not contain dedicated tools for water quality modeling and mapping, besides their high cost and inaccessibility in resource-limited regions. While some remote sensing aquatic applications software packages like NASA’s SeaDAS (SeaWiFS Data Analysis System) are valuable, they are typically standalone applications focusing on specific tasks (e.g., SeaDAS specializes in ocean color processing of MODIS/VIIRS data). Moreover, reproducibility across studies remains a persistent challenge because processing decisions—such as atmospheric correction, masking criteria, or model calibration choices—are frequently undocumented or difficult to replicate (Palmer et al. 2015; Dörnhöfer and Oppelt 2016).

From a methodological perspective, three persistent barriers have limited broader adoption of aquatic remote sensing workflows:

1. Atmospheric correction in optically complex waters. The presence of high concentrations of dissolved and suspended constituents leads to non‑linear interactions between water‑leaving and atmospheric signals. This complexity makes the accurate retrieval of surface reflectance significantly more challenging than in clear, open-ocean (Case-1) waters (Odermatt et al. 2012). While dedicated tools such as ACOLITE, POLYMER, and SeaDAS support water‑specific correction schemes, their use outside ocean‑color communities remains technically demanding (IOCCG 2000; Vanhellemont and Ruddick 2018; Pahlevan et al. 2021).
2. Algorithm transferability and generalization. Empirical and semi‑analytical algorithms trained for one optical regime often degrade in others, necessitating site‑specific recalibration. Machine‑learning methods can mitigate this but raise issues of overfitting and interpretability (Matthews 2011; Spyrakos et al. 2018; Politi et al. 2015).
3. Computational scalability and usability. Processing multi‑temporal imagery requires substantial computational resources. Scripts written for research prototyping seldom support multi‑threading, memory‑bounded processing, or integration with interactive geographic information systems (GIS) (Harris et al. 2020; Gillies 2013; Jordahl et al. 2020).

The open‑source GIS platform QGIS offers a promising foundation for addressing these barriers due to its robust Python API, large user community, and plugin ecosystem; yet most tools focus on generic raster processing rather than domain‑specific applications such as water‑quality retrieval (Renard et al. 2024). A specialized, end‑to‑end solution that embeds proven aquatic algorithms into QGIS could therefore bridge the gap between research‑grade methods and operational workflows.

We present RS‑WaterQuality Mapper, a generalizable, open‑source framework implemented as a QGIS plugin that integrates the end‑to‑end workflow within a single, GIS‑native environment. Conceptually, the framework formalizes eight modelling abstractions: (1) sensor‑agnostic data ingestion; (2) water/land/cloud masking with interactive and automated options; (3) aqua‑focused atmospheric correction tailored to inland/coastal waters; (4) feature construction via indices and band ratios; (5) matchup generation that pairs in situ measurements with image neighborhoods under mask awareness; (6) estimation using empirical regressions, machine‑learning models (e.g., Random Forest, Support Vector Regression), and a selective ensemble strategy; (7) validation with cross‑validation/hold‑out testing and uncertainty summaries; and (8) visualization and export of GIS‑ready products for analysis, reporting, and downstream model coupling.

To test generalizability, we evaluate the framework on three contrasting systems that span common inland‑water challenges: (i) a bloom‑prone U.S. reservoir where red‑edge information is critical for chlorophyll‑a; (ii) a tropical saline lake with strong productivity and shoreline effects, requiring careful masking and multi‑parameter retrieval; and (iii) a river confluence with contrasting sediment regimes where turbidity retrieval must contend with adjacency and narrow channel geometry (Kuhn et al. 2019). These cases are not presented as isolated showcases but as experiments probing how modelling choices and uncertainties play out under different optical and geomorphic conditions.

The remainder of this article is organized as follows: Sect. 2 positions this work relative to existing toolchains. Section  3 details the framework and architecture. Section 4 presents case studies illustrating performance across diverse optical and hydrological regimes. Section  5 synthesizes generalizable insights, limitations, and future directions, and Sect.  6 concludes.

## Related work & positioning

### Existing toolchains and gaps

A range of capable tools support pieces of workflow. Mission‑specific or ocean‑color‑oriented processors provide robust atmospheric correction and Level‑2 products but are often standalone and optimized for open‑ocean/coastal conditions rather than inland waters (Doron et al. 2011; Vanhellemont and Ruddick 2018). General remote‑sensing suites and cloud platforms offer powerful computation and data access—e.g., Google Earth Engine implementations for lake monitoring—but can lead to fragmented pipelines assembled from custom scripts. Crucially, GEE currently lacks native, appropriately atmospherically corrected products for water use; terrestrial-focused surface reflectance products often fail to account for the unique optical properties of inland waters, requiring users to implement complex, external correction algorithms (Wang et al. 2020; Kislik et al. 2022). Furthermore, these platforms are not always practical for offline or regulated environments (Topp et al. 2020). Within the GIS ecosystem, users benefit from strong visualization and cartography, yet there are comparatively few GIS‑native, open‑source toolchains that carry users from ingestion through correction, masking, feature construction, modelling, validation, and export in a single, auditable environment (Renard et al. 2024).

These gaps manifest for practitioners as (i) a steep barrier to entry for non‑programmers; (ii) duplicated effort when each team reimplements similar pipelines; (iii) difficulty in comparing methods and communicating uncertainty; and (iv) challenges linking outputs to management workflows (e.g., thresholds, alerts, and reporting) within familiar GIS (Palmer et al. 2015; Dörnhöfer and Oppelt 2016; Topp et al. 2020).

### How this work advances the state of practice

Concretely, this work contributes:

- A formalized framework that makes the workflow components and their interactions explicit and auditable inside a widely adopted GIS (Renard et al. 2024).
- A comparative evaluation protocol including single‑index baselines, machine learning alternatives, ensemble strategies, and uncertainty summaries, enabling apples‑to‑apples comparisons across sites and targets (Keller et al. 2018; Ma et al. 2021; Fang et al. 2022).
- Robust matchup generation and mask-aware sampling that address adjacency and mixed pixels—recurring pain points in lakes and rivers (Kutser et al. 2005).
- A decision-support orientation linking products to management-relevant thresholds and reports, and facilitating data exchange with process models (Carlson 1977; Watanabe et al. 2016).

In combination, these advances aim to lower the barrier from research prototypes to operational monitoring, while preserving scientific rigor and transparency expected by the environmental modelling and software community.

## Framework & architecture

### Design principles

The overall design of RS-WaterQuality Mapper follows these guiding principles:

- **Genericity & Sensor-Agnosticism.** Support decameter multispectral sensors (e.g., Sentinel-2 MSI, Landsat-8/9 OLI) and allow future extension to additional sensors (including hyperspectral) via declarative band maps and feature builders.
- **End-to-End Modelling.** Provide a seamless path from imagery ingestion to atmospheric correction, masking, feature extraction, modelling, and visualization - within a single GIS-native environment.
- **Modularity and Extensibility.** Each processing component (masking, atmospheric correction, feature extraction, modelling, visualization) is implemented as a self-contained class following QGIS plugin standards. Users can run a complete workflow or call individual modules as needed.
- **Collaborative Development & Facilitation.** Designed for wide-scale adoption through open-source licensing and transparent version control. By providing source codes and clear documentation, the tool facilitates community contributions, ensuring that local adaptations can be shared and integrated back into the global pipeline.
- **Usability for Non-Programmers.** Offer a guided interface with sensible defaults and clear prompts while preserving expert controls for advanced users.
- **Performance & Robustness.** Handle moderately large rasters efficiently; isolate long-running jobs off the UI thread; and fail gracefully with informative diagnostics.
- **Interoperability.** Read/write standard geospatial formats (GeoTIFF and shapefile), integrate with external correction tools, and export products consumable by other modelling systems and dashboards.
- **Targets Decision Support.** Outputs are designed for thresholding (e.g., trophic state classes), hotspot identification, and coupling with process models and dashboards.

### Modular architecture and data flow

**Fig. 1:**
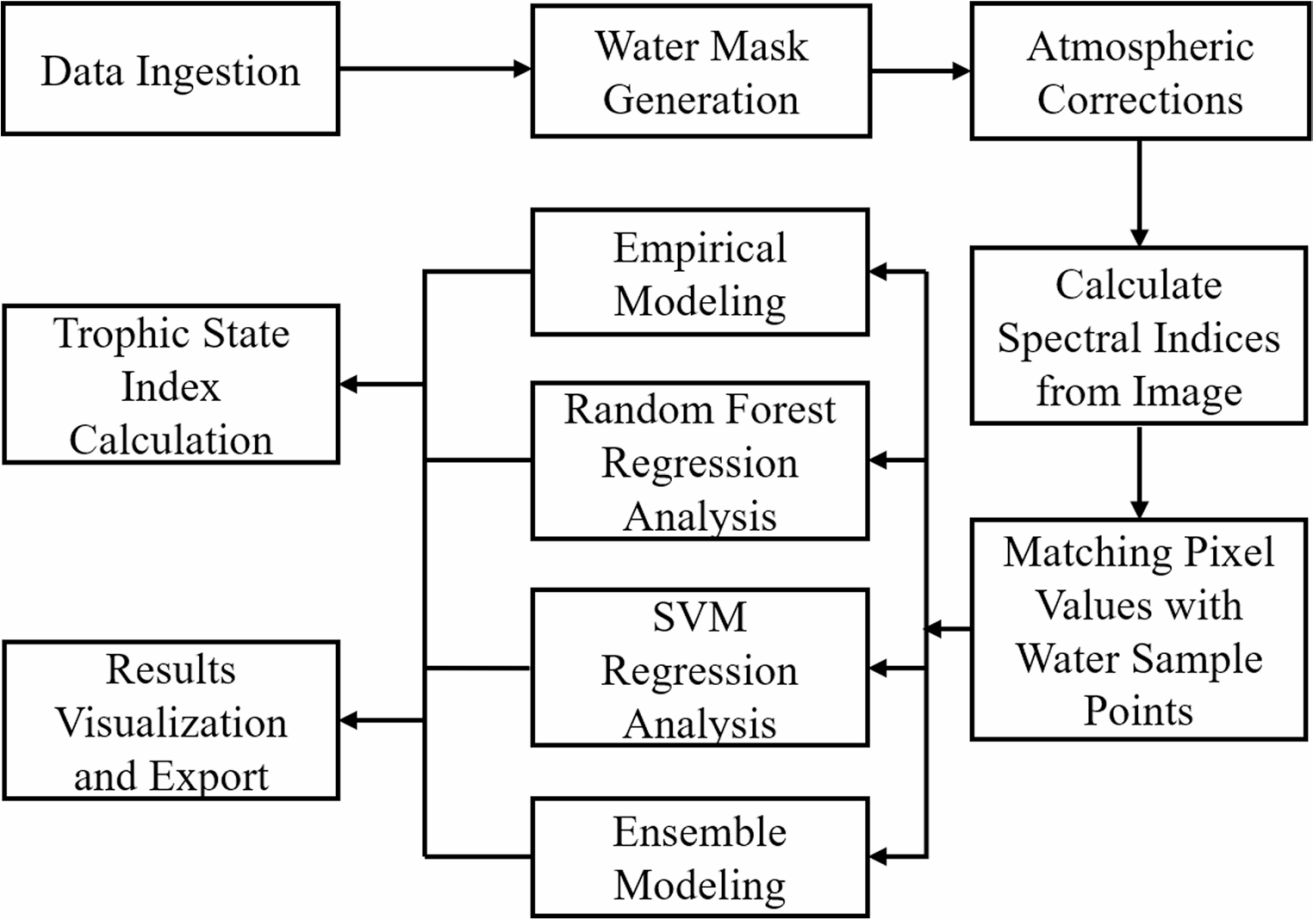
Water quality remote sensing modeling and mapping workflow

**Fig. 2:**
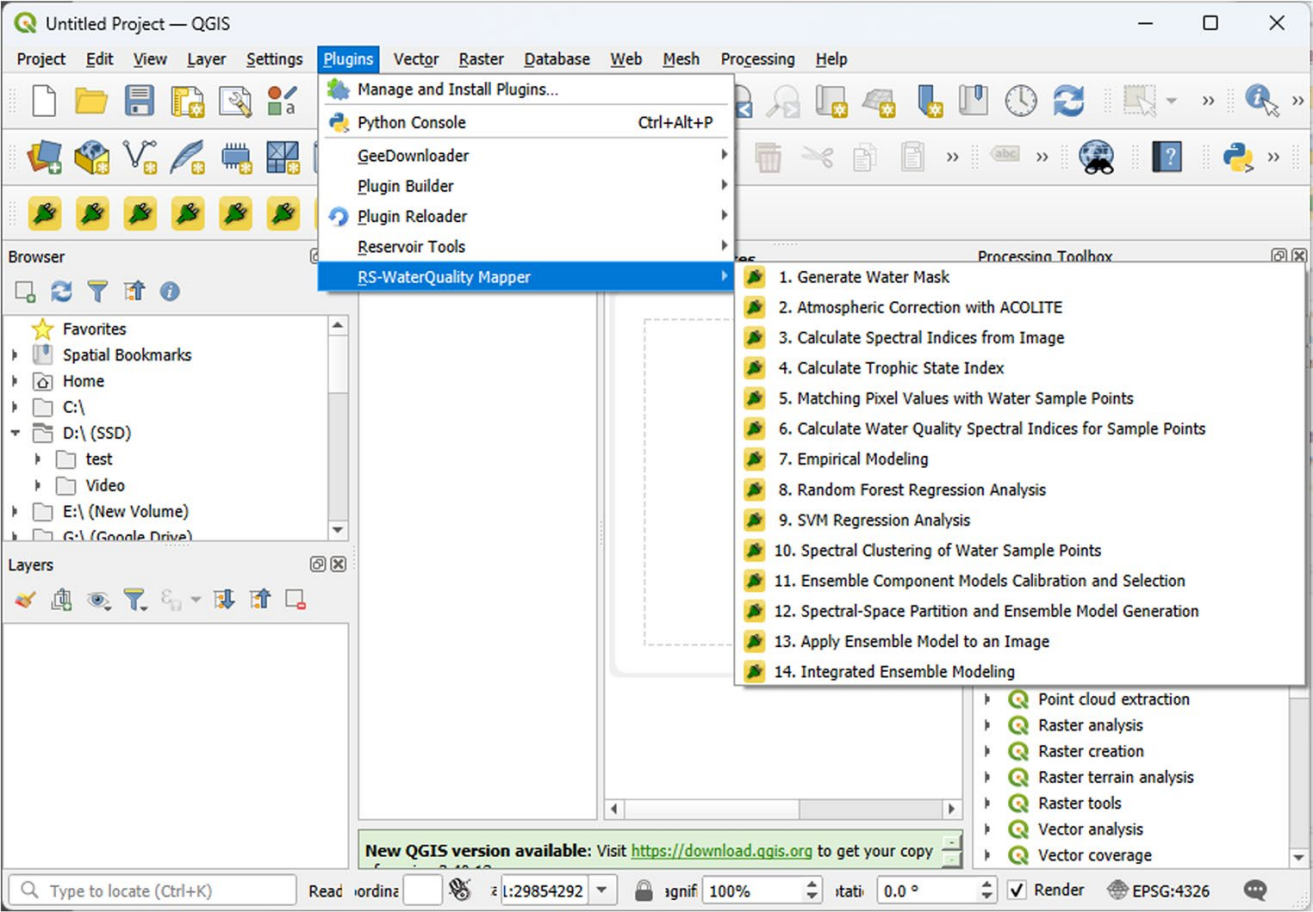
RS‑WaterQuality Mapper QGIS plugin interface

RS‑WaterQuality Mapper is organized into interacting modules. A typical session activates modules in the order below (Fig. 1); however, modules can be invoked independently when prior steps are performed elsewhere. To facilitate analysis over time, the toolbox supports the sequential processing of multiple satellite datasets. It is important to note that the current version of the plugin operates on a scene-by-scene basis. Users are required to load and process each satellite image individually through the graphical user interface (Fig. 2).

1. Data Ingestion

The RS-WaterQuality Mapper is designed to process pre-downloaded, locally stored satellite imagery. Users acquire Level-1 or Level-2 products from standard data providers (e.g., USGS EarthExplorer, Copernicus Data Space Ecosystem) and save them to a local directory.

The “ingestion” process is fully automated within the plugin to eliminate manual data preparation. Users simply navigate to the input directory (e.g., a Sentinel-2 .SAFE folder or a Landsat product folder) via the GUI. The plugin currently supports Sentinel-2 MSI and Landsat-8/9 OLI products. It utilizes declarative band maps, internal definitions that map sensor-specific file naming conventions to standard spectral channels (e.g., Blue, Green, Red, NIR), allowing the software to identify and read the necessary bands using the Rasterio library without manual layer stacking. This architecture allows for future extension to new sensors, such as Sentinel-3 OLCI or PACE, by simply updating the internal feature-definition scripts without altering the core codebase.

2. Water Masking

Accurate isolation of valid water pixels is a prerequisite for robust modeling. The toolbox implements a multi-tiered masking strategy to ensure reliability:

- Spectral Thresholding. Water pixels are identified using the Normalized Difference Water Index (NDWI) or near-infrared thresholds (McFeeters 1996). An adaptive Gaussian-mixture model allows users to visually calibrate the cutoff for specific scenes (Liu and Jezek 2004).
- Geometric Buffering. To mitigate edge-mixing effects, users can apply a negative buffer to vector-based lake or river boundaries, physically eroding the mask to exclude the “shoreline mixing zone” where land and water signals are inextricably mixed (Kutser et al. 2005).
- Cloud and Shadow Management. To manage atmospheric contamination, the toolbox ingests the standard Quality Assessment (QA) layers provided by the data providers (e.g., the Scene Classification Layer (SCL) for Sentinel-2 or the Pixel Quality Assessment (QA_PIXEL) band for Landsat 8/9). These layers are used to strictly mask out clouds, cirrus, and crucially cloud shadows, which typically introduce severe errors in dark-target water retrieval (Zhu & Woodcock, 2012).

Masks generated by these methods can be combined through logical operations (union, intersection) depending on user preference. The final mask is stored as a GeoTIFF for reuse in subsequent processing.

3. Aqua-Focused Atmospheric Correction

Accurate retrieval of surface reflectance in optically complex inland and coastal waters (often referred to as Case-2 waters) is significantly more challenging than in clear, open-ocean environments. Unlike ocean waters where optical properties are primarily determined by phytoplankton, Case-2 waters contain complex, independent mixtures of chlorophyll-a, suspended sediments, and Colored Dissolved Organic Matter (CDOM). These constituents create non-linear interactions between water-leaving radiance and atmospheric signals, often leading to standard atmospheric correction algorithms failing due to low signal-to-noise ratios and the presence of dark targets. To address the challenges inherent in Case-2 waters, the toolbox incorporates ACOLITE, a specialized atmospheric correction processor. To manage ACOLITE’s external dependencies and configuration complexity, the plugin utilizes a modular interface where users simply specify the path to the ACOLITE launch.py script and the desired input/output directories.

The plugin automates the generation of the necessary settings files for ACOLITE’s Dark Spectrum Fitting (DSF), RAdCor and Exponential (EXP) algorithms (Vanhellemont and Ruddick 2018). Notably, the integration of the RAdCor processor allows the system to specifically address adjacency effects in atmospheric correction by modeling the scattering contribution from bright nearby land targets. For advanced users, the interface allows for the ingestion of a custom settings file, enabling full control over ACOLITE’s extensive parameter set. If no custom setting file is provided, the plugin applies sensible defaults that automatically trigger the generation of surface reflectance products in GeoTIFF format alongside the standard NetCDF files. By streamlining this execution through system calls and automated parameter mapping, the toolbox provides a reliable path for retrieving water-leaving reflectance in optically complex water bodies without requiring manual command-line intervention.

4. Feature Construction

Following atmospheric correction, RS-WaterQuality Mapper constructs a library of spectral features used for empirical and machine-learning retrievals. Features include:

- Raw reflectance bands.
- Band ratios and differences (e.g., Blue/Green, Red/NIR).
- Published spectral indices such as the Normalized Difference Chlorophyll Index (NDCI) (Mishra and Mishra 2012), the Normalized Difference Turbidity Index (NDTI) (Nechad et al. 2010; Dogliotti et al. 2015), and the Blue–Green Ratio (BGR) for CDOM proxies.

The toolbox is designed to be dynamically extensible, allowing for the rapid addition of new spectral features as research evolves. Because the feature construction module utilizes a declarative mapping system, users can integrate new indices, such as hyperspectral-derived metrics or emerging Case-2 water algorithms, by simply updating the internal feature-definition scripts. This “open-code” architecture ensures that the plugin remains a flexible research tool, capable of adapting to new sensor specifications or localized water quality proxy requirements without necessitating a fundamental redesign of the core processing engine.

5. Matchup Generation

To pair in situ observations with satellite imagery, the toolbox extracts reflectance and derived features from a 3*3 or 5*5 pixel window centered on each sampling station by computing robust statistics (e.g., the median) across water pixels within the window. The resulting output is a synchronized table including: sample ID, timestamp, coordinates, response variable(s), predictors, and quality flags.

6. Empirical and Machine-Learning Models

The modelling module supports both classical and modern regression approaches, providing a consistent interface for parameter estimation and evaluation. The implemented methods include:

- Linear regression (LR) for rapid empirical relationships.
- Random Forest (RF) and Support Vector Regression (SVR) for nonlinear relationships. The use of RF, a powerful ensemble method based on decision trees (Breiman 2001), and SVR has become increasingly common for water quality remote sensing due to their ability to capture complex, non-linear relationships (Keller et al. 2018; Li et al. 2017; Pal and Mather 2003).
- Spectral-space partitioned ensemble (SPE), a hybrid strategy developed for this toolbox.

The SPE approach represents a key scientific contribution. It was developed as a direct response to the well-documented problem of poor model transferability across different optical water types (Politi et al. 2015). It recognizes that optical diversity across water bodies often leads to heteroscedastic residuals in global models. To address this, predictor space (reflectance or indices) is segmented into quasi-homogeneous regions using decision-tree partitioning. Within each region, candidate models (e.g., RF, SVR, LR) are trained separately. At prediction time, the input spectrum is routed to the appropriate submodel according to its partition membership. A central innovation of this approach lies in using the spectral feature space as a surrogate for aquatic environmental variability. By partitioning the spectral domain, the ensemble model effectively captures variations in aquatic optical properties and assigns the most suitable component model to each spectral region. This selective modeling strategy leverages the comparative strengths of multiple empirical models, allowing the ensemble to adapt dynamically to a wide range of aquatic environments. As a result, the approach offers superior generalization and transferability across diverse geographic regions and temporal conditions, overcoming the limitations of conventional site-specific empirical models that often lack robustness when applied beyond their original calibration context (Politi et al. 2015). Experimental results have demonstrated that the ensemble model not only improves predictive performance but also exhibits strong spatial transferability and seasonal consistency (Xu et al. 2021, 2023; Liu et al. 2024).

All models are trained using scikit-learn, ensuring numerical robustness and portability (Pedregosa et al. 2011). Cross-validation (leave-one-out) is used to estimate out-of-sample performance. Standard error metrics (R², RMSE, MAE) are reported automatically and stored in metadata logs.

7. Mapping & Export

After model training, predictions are applied to full images through a rasterized mapping function. To maintain computational efficiency, rasters are processed block-wise, typically in 512 × 512 pixel windows. Generated concentration maps are automatically symbolized in QGIS using color ramps appropriate for each parameter (e.g., chlorophyll-a in mg m⁻³, turbidity in NTU). Users can optionally classify outputs into trophic or turbidity categories using standard thresholds (Carlson 1977; Watanabe et al. 2016).

### Computational efficiency

The plugin employs a suite of robust, high-performance open-source Python libraries, creating a transparent “glass box” architecture that promotes reproducibility and trust. This design stands in contrast to proprietary “black box” systems, as every component of the processing chain is built upon community-vetted, open-source code. The core libraries include Rasterio for raster I/O (Gillies 2013), GeoPandas for vector operations (Jordahl et al. 2020), NumPy for numerical computations (Harris et al. 2020), and scikit-learn for machine learning (Pedregosa et al. 2011). Citing and building upon these foundational projects is a declaration of alignment with the open-science movement.

A key design consideration was ensuring computational efficiency when processing large satellite scenes. This is achieved through four primary strategies:

- **Selective Processing**: Operations like spectral index calculations and modeling are restricted to water-masked pixels, reducing computational load.
- **Multi-threading**: Parallel processing of image tiles and model training minimizes runtime, leveraging multi-core CPUs.
- **Memory-Efficient Data Handling**: The toolbox leverages the block-based processing capabilities of Rasterio. Instead of loading entire multi-gigabyte files into memory, data is read, processed, and written in smaller chunks, enabling the analysis of full satellite scenes on standard computers without exceeding available RAM.
- **Optimized Backend**: Rasterio and NumPy are built on compiled C/C + + code, while scikit-learn’s algorithms are tuned for sparse data handling. Reliance on these libraries ensures that core calculations on large data arrays are performed rapidly. For example, processing a 10,000 × 10,000-pixel Sentinel-2 image with 10 bands typically completes index calculations in < 30 s and ensemble model training in < 2 min on a standard desktop (8-core CPU, 16 GB RAM). These optimizations ensure scalability for large-scale monitoring.

## Application examples

To demonstrate the capabilities and validate the performance of RS-WaterQuality Mapper, the toolbox was applied to three diverse case studies representing different water body types and geographical settings.

### Harsha Lake (Ohio, USA)

Harsha Lake is a mesotrophic to eutrophic reservoir in southwest Ohio, USA (~ 8 km²) that experiences periodic harmful algal blooms (HABs). HAB risk and eutrophication dynamics motivate quantitative chlorophyll‑a mapping for management interventions (Paerl and Huisman 2008; Paerl and Paul 2012). This case study focused on mapping chlorophyll-a during a bloom event using a Sentinel-2 A image from October 7, 2016, which was concurrent with an in-situ sampling campaign that collected 56 measurements. The Chl-a values ranged from ~ 8 to ~ 87 µg/L, indicating a significant bloom.

**Fig. 3:**
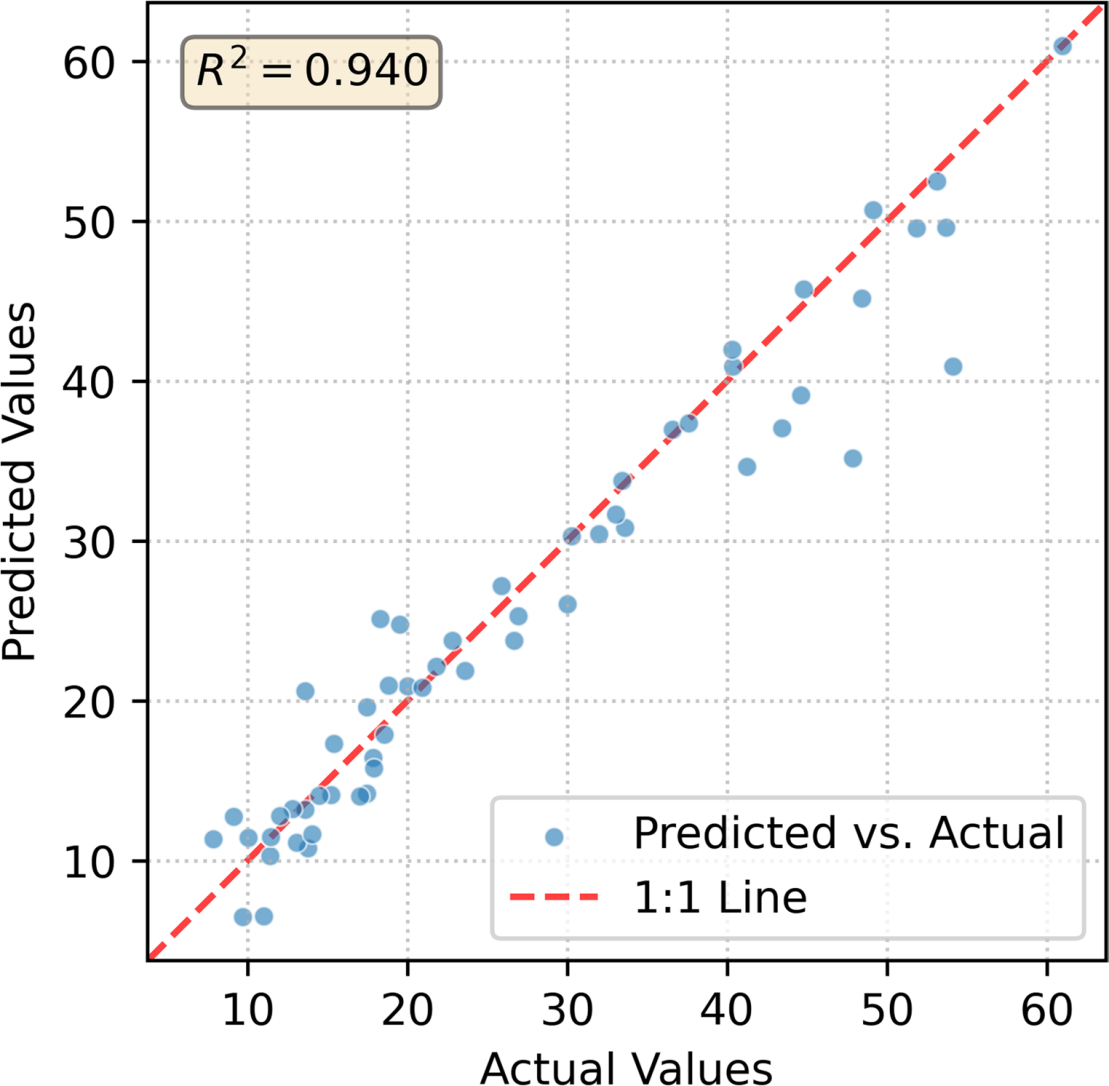
Scatter plot shows the performance of ensemble model for Chlorophyll-a

Within the toolbox, a water mask was generated using the Normalized Difference Water Index (NDWI) and refined with the Sentinel-2 Scene Classification Layer to isolate the lake surface and exclude clouds. The image was then atmospherically corrected using the ACOLITE Dark Spectrum Fitting algorithm. The Normalized Difference Chlorophyll Index (NDCI) was computed, and the 56 field measurements were used to develop a multi-predictor ensemble model. The resulting SPE model demonstrated high predictive performance for chlorophyll-a, as shown in Fig. 3.

**Fig. 4:**
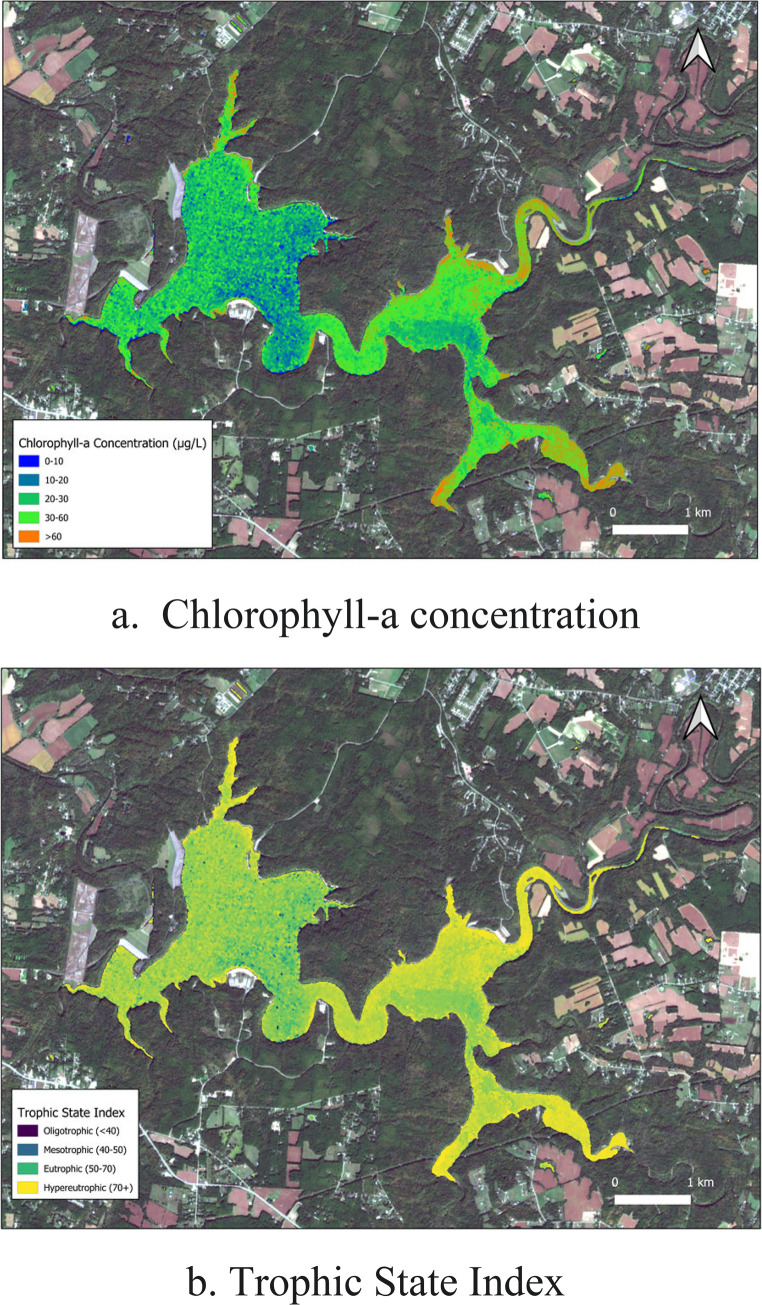
Chlorophyll-a concentration and trophic state index maps of Harsha Lake produced by the QGIS toolbox

The SPE model was applied to the entire scene, producing a quantitative chlorophyll-a concentration map (Fig. 4a). The map accurately delineated the spatial extent and magnitude of the algal bloom, with predicted concentrations aligning well with ground-truth data (R2 ≈ 0.94, RMSE = 3.73 µg/L). Using the plugin’s Trophic State Index Calculation module, we converted the retrieved Chlorophyll-a concentrations into TSI values based on Carlson’s standard equations. We then applied custom thresholds to categorize the reservoir into Mesotrophic, Eutrophic, and Hypereutrophic zones (Fig. 4b). This application demonstrates the toolbox's effectiveness in providing valuable, quantitative information for local water managers monitoring episodic blooms in small reservoirs.

### Lake Nakuru (Kenya)

To demonstrate global applicability, the toolbox was used to assess Lake Nakuru, a small, highly saline, and productive alkaline lake in the African Rift Valley. The lake is prone to massive cyanobacteria blooms and is a RAMSAR Wetland of International Importance. A Sentinel-2 image from June 10, 2023, was used, coinciding with a field campaign that collected 33 surface samples for chlorophyll-a, turbidity, and fluorescent dissolved organic matter (fDOM).

**Fig. 5:**
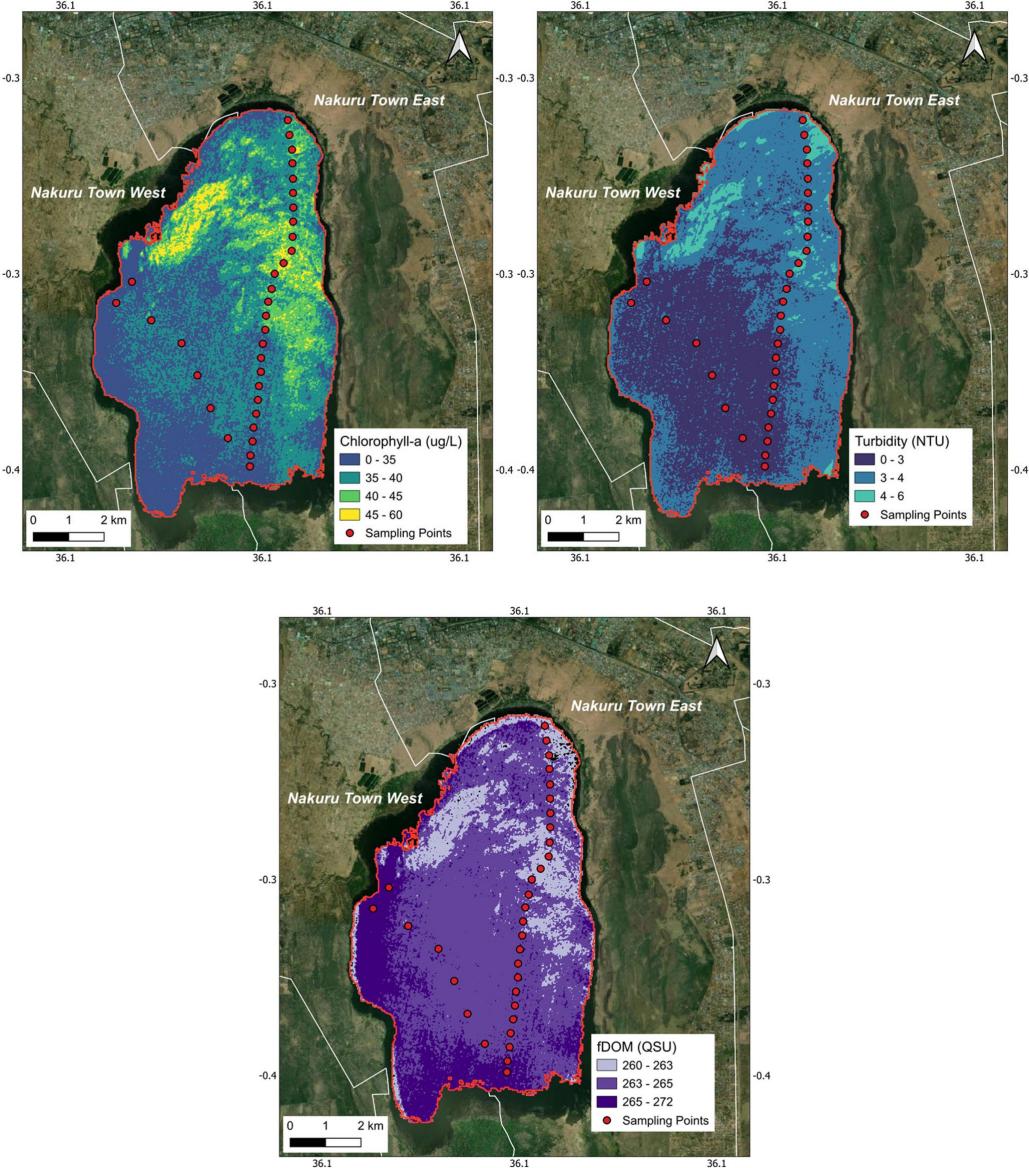
Chlorophyll-a concentration, turbidity, and fDOM maps of Lake Nakuru produced by the QGIS toolbox

Following atmospheric correction with ACOLITE, a water mask was generated using an interactive optimal thresholding approach on the NDWI. An ensemble modeling strategy was employed for each water quality parameter. Multiple empirical models using different spectral indices (e.g., NDCI, NDTI, Red/Blue ratio) were calibrated with the field data and then combined. The ensemble models achieved high performance on the training data for chlorophyll-a (R^2^ ≈ 0.91, RMSE = 1.73) and turbidity (R^2^ ≈ 0.92, RMSE = 0.15), successfully capturing the strong gradients in algal biomass and suspended solids characteristic of this hypereutrophic system. However, the retrieval accuracy for fDOM was notably lower (R^2^ ≈ 0.77, RMSE = 1.18). This disparity is attributed to the specific hydro-chemical conditions observed during the sampling campaign. While Lake Nakuru exhibited extremely high baseline fDOM concentrations, the spatial variance within the sampled dataset was remarkably low, with values clustered tightly between 261 and 265 QSU (Quinine Sulfate Units). This narrow dynamic range limits the statistical power of regression analysis, as the signal variance is small relative to potential sensor noise and atmospheric residual errors. Consequently, while the resulting map (Fig. 5) visualizes the general distribution of dissolved organic matter, we caution that the model for this specific parameter under these specific conditions is less robust than those for particulate constituents. Nevertheless, Fig. 5 revealed distinct spatial patterns, such as a chlorophyll hotspot in the northeast corner coinciding with high turbidity, likely indicating a surface scum. This case study showcases the toolbox’s utility in a data-sparse region, enabling local scientists and park managers to gain a synoptic view of their lake’s condition for conservation and monitoring.

### Tombigbee river and black warrior river (Alabama, USA)

The final case study demonstrates the toolbox’s utility for riverine monitoring, focusing on mapping turbidity at the confluence of the Tombigbee and Black Warrior rivers. A Landsat 8 OLI scene from May 2, 2019, was used, coordinated with a boat-based survey that collected 57 turbidity measurements. A water mask was generated for the rivers using an optimal NDWI threshold and refined using the Landsat 8 Quality Assessment (QA) band to remove extensive cloud cover.

**Fig. 6:**
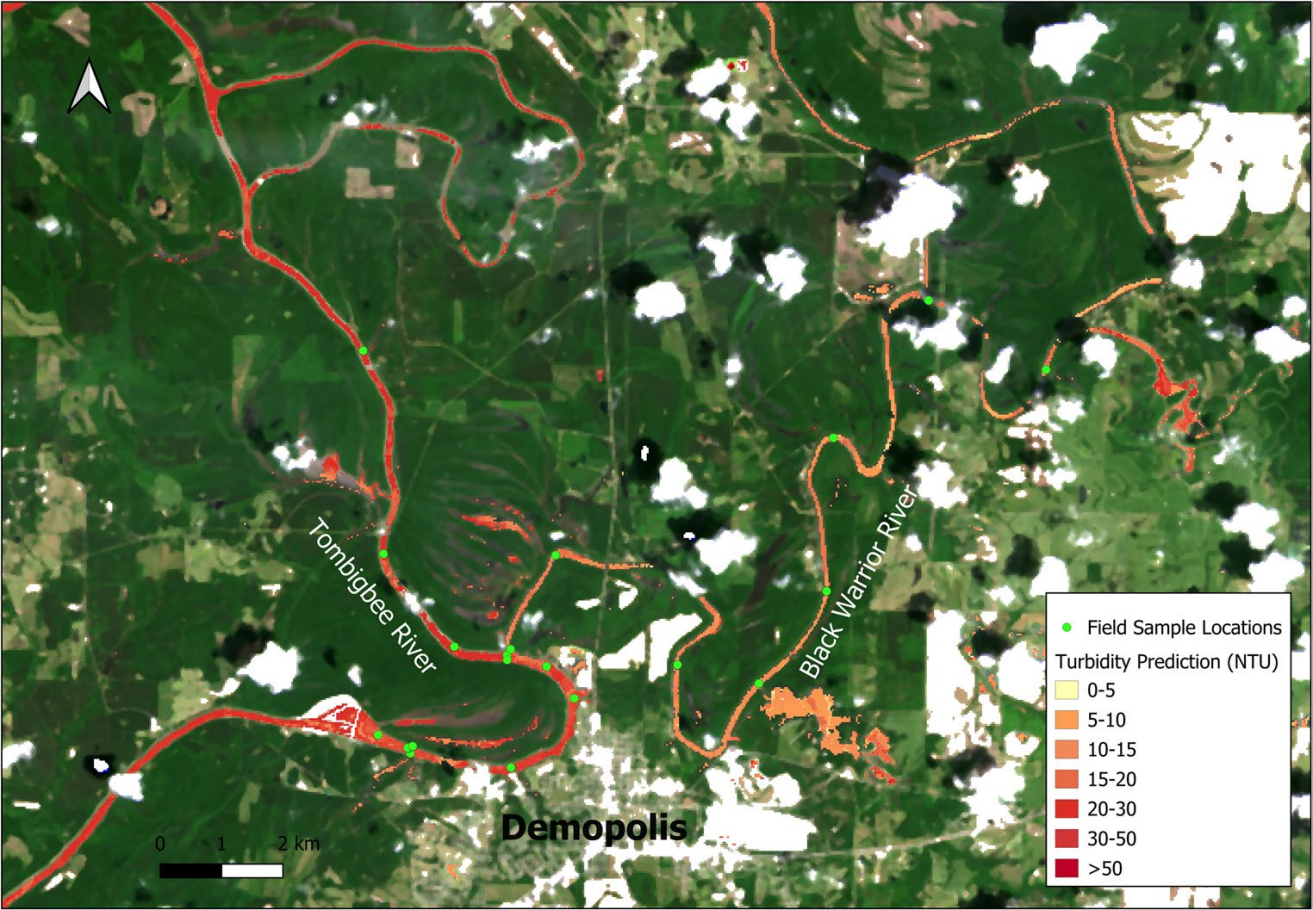
Field sampling sites near Demopolis, Alabama and turbidity prediction map from ensemble model

Given the narrow channel geometry of the river system and the proximity of dense riparian vegetation, the imagery is highly prone to adjacency effects, where radiance reflected from bright land targets scatters into the sensor’s field of view over darker water pixels. To address this, atmospheric correction was performed using the RAdCor processor within the ACOLITE framework. Unlike standard methods that assume a uniform atmosphere, RAdCor specifically addresses adjacency effects by modeling the scattering contribution from nearby land targets. This radiometric correction allowed for the recovery of valid water-leaving reflectance spectra closer to the banks than would be possible with simple geometric buffering, maximizing the observational width of the river channel. Four different turbidity-related indices (NDTI, Red/Blue ratio, etc.) were calculated and used as predictors in an ensemble model calibrated with the field data. The ensemble model yielded a significantly better fit than any single index (R^2^≈ 0.80) and was applied to produce a turbidity map of the river system (Fig. 6). The resulting map clearly delineated the higher turbidity of the Tombigbee River compared to the clearer Black Warrior River, with the patterns matching field observations. This application highlights the toolbox’s flexibility for monitoring linear, dynamic river systems, providing valuable insights for sediment management and environmental assessment.

As shown in Table 1, across the three case studies presented, a small eutrophic lake, a large tropical alkaline lake, and a dynamic river system, the RS-WaterQuality Mapper toolbox consistently produced reliable water quality maps. These outputs were validated by in situ data and known environmental patterns, reinforcing the potential of integrating satellite remote sensing within QGIS to provide scientifically credible and operationally useful information for diverse aquatic environments.

**Table 1:** Summary of in situ datasets and ensemble model performance metrics generated by the toolbox

Case Study	Sensor	Target Parameter	*N*	Range	*R* ^2^	RMSE
Harsha Lake	Sentinel-2	Chlorophyll-a (µg/L)	56	8.0–87.0	0.94	3.73
Lake Nakuru	Sentinel-2	Chlorophyll-a (µg/L)	33	25.0–62.0	0.91	1.73
Lake Nakuru	Sentinel-2	Turbidity (NTU)	33	2.0–8.0	0.92	0.15
Lake Nakuru	Sentinel-2	fDOM (QSU)	33	261–265	0.77	1.18
Tombigbee River	Landsat 8	Turbidity (NTU)	57	8.7–57.5	0.8	4.94

## Discussion

The development of the RS-WaterQuality Mapper represents a deliberate effort to bridge the persistent and well-documented gap between the scientific advancements in aquatic remote sensing and their operational implementation in water resource management. This discussion synthesizes the results from the diverse case studies to evaluate the toolbox’s success in this endeavor, contextualizes its key methodological innovations within the current state of the practice, explores the broader implications for global water monitoring and collaborative science, and critically assesses its limitations to define a clear path for future development.

### An integrated framework for bridging the research-to-operations gap in water quality monitoring

A central challenge hindering the widespread adoption of satellite-based water quality monitoring has been the fragmented nature of existing workflows, which often require practitioners to navigate multiple software environments, command-line utilities, and custom scripts. This fragmentation creates significant technical barriers, compromises reproducibility, and disconnects the scientific process of data analysis from the management context of decision-making. The RS-WaterQuality Mapper was designed not merely as a collection of processing tools, but as a cohesive and integrated *framework* that directly addresses these systemic issues. The successful application of the toolbox across three optically and geomorphologically distinct aquatic systems provides compelling evidence that this integrated approach is both effective and generalizable.

The case studies were not selected as isolated showcases of performance but were strategically chosen as a rigorous “stress test” to probe the framework’s robustness under challenging, real-world conditions that often cause specialized algorithms to fail. In Harsha Lake, a temperate U.S. reservoir experiencing a significant algal bloom with chlorophyll-a concentrations reaching approximately 87 µg/L, the toolbox demonstrated exceptional predictive power (R^2^ ≈ 0.94). This result validates the framework’s utility for a critical management application: the quantitative mapping of eutrophication and harmful algal blooms, a pressing issue in inland waters worldwide. The application in Lake Nakuru, a highly productive and saline tropical lake in a data-sparse region of Kenya, showcased the framework’s global applicability and its capacity for robust multi-parameter retrieval (chlorophyll-a, turbidity, and fDOM) with strong performance (R^2^ ≈ 0.90) in an extreme and optically complex environment. Finally, the successful mapping of turbidity in the Tombigbee and Black Warrior River system in the U.S. demonstrated the framework’s effectiveness in challenging riverine settings, where factors like narrow channel geometry, shoreline adjacency effects, and high sediment loads complicate remote sensing analysis.

The consistent high performance across this spectrum of environments, from a bloom-prone temperate reservoir to a productive tropical saline lake and a dynamic temperate river system, is not coincidental. It is a direct validation of the framework’s core design philosophy: to provide an end-to-end, scientifically sound workflow within a single, GIS-native environment. By seamlessly integrating aqua-focused atmospheric correction via ACOLITE, robust masking procedures, advanced model training, and the generation of GIS-ready map products, the toolbox removes the procedural friction that typically plagues such analyses. This integration fosters a fundamental paradigm shift in how water quality remote sensing is conducted. It moves the user away from a piecemeal data processing mentality toward a more holistic and integrated modeling approach. Within this framework, critical decisions made at each step, such as the choice of a masking buffer or an atmospheric correction algorithm, are part of a transparent and auditable sequence, forcing the user to consider how early choices propagate uncertainty through to the final map product. This conceptual reframing elevates the scientific rigor of the analysis, promoting better science, not just easier processing.

### Advancing methodological rigor and accessibility in aquatic remote sensing

Beyond its integrated design, the RS-WaterQuality Mapper advances the state of the practice by embedding key methodological innovations that enhance both scientific rigor and accessibility for a broader user community. Two contributions are particularly significant: the implementation of the spectral-space partitioned ensemble (SPE) model and the deliberate effort to make the entire workflow transparent, reproducible, and usable by non-specialists.

A primary scientific challenge in aquatic remote sensing is the poor transferability of algorithms; models calibrated for one optical regime frequently exhibit degraded performance when applied to different locations or seasons. The SPE model implemented in this toolbox addresses this by mitigating the overfitting often observed in global machine-learning models. The robustness of this approach has been rigorously established in prior studies using the datasets presented here. For the Harsha Lake dataset, Xu et al. (2021) demonstrated both spatial and temporal extensibility: the SPE model calibrated on Harsha Lake (2016) was successfully transferred to Caesar Creek Lake and Brookville Lake on the same date (Spatial), and to Harsha Lake in 2017 and 2018 (Temporal) without recalibration. In both cases, the ensemble model significantly outperformed traditional algorithms, achieving an R^2^ of 0.89 for temporal prediction and effectively mapping distinct lakes with different optical properties. Similarly, for the Tombigbee River case study, Xu et al. (2023) validated the spatial transferability of the method at a basin scale, showing that the model maintained high performance (R^2^ =0.93) when transferred across three distinct sub-basins with varying sediment regimes. Therefore, while the maps presented in Sect.  4 serve to demonstrate the software workflow (ingestion to mapping), the underlying SPE engine has been independently verified to support the spatiotemporal extensibility required for operational monitoring. This architecture allows the framework to adapt to diverse optical water types without requiring the user to manually classify water bodies.

Simultaneously, the toolbox is designed to lower the formidable barrier to entry that has historically limited the use of remote sensing to a small community of experts. By embedding a complex process like water-specific atmospheric correction with ACOLITE into a guided, menu-driven interface, the toolbox makes a critical and technically demanding step accessible to users outside the traditional ocean-color community. This design philosophy provides what can be described as “methodological scaffolding” for non-experts. The toolbox does not simply provide a set of functions; its structured workflow and provision of “sensible defaults” guide the user through a scientifically sound analysis process. This embedded expertise helps prevent common but critical errors, such as improper masking of land and clouds or ignoring shoreline adjacency effects, thereby elevating the quality and reliability of analyses performed by a wider range of practitioners. Furthermore, by being fully open-source with an auditable record of all configurations and outputs, the toolbox stands in stark contrast to proprietary “black box” solutions. This transparency directly addresses the persistent challenge of reproducibility in the field, allowing for full inspection, validation, and modification of its methods, which builds trust and confidence in scientific outputs. The emphasis on computational efficiency, achieved through multi-threading and block-based processing with libraries like Rasterio, is a crucial enabling feature, ensuring that the analysis of entire satellite scenes is feasible on standard desktop computers, a prerequisite for any tool intended for routine operational use.

### Broader implications for global water management and collaborative science

The true impact of the RS-WaterQuality Mapper extends beyond its technical and methodological contributions to its potential to transform water resource management and foster a more collaborative scientific culture on a global scale. By strategically democratizing access to advanced satellite data analytics, the toolbox empowers a diverse community of stakeholders to address pressing water quality challenges, particularly in regions where ground-based monitoring is sparse or non-existent.

The Lake Nakuru case study serves as a powerful narrative anchor for this potential impact. In many parts of the world, particularly across Africa, Asia, and Latin America, the logistical and financial constraints of maintaining extensive in-situ monitoring networks leave vast gaps in our understanding of water quality dynamics. The successful application of the toolbox in Kenya demonstrates its capacity to fill these observational gaps, enabling local scientists, conservationists, and park managers to gain a synoptic, near-real-time view of their aquatic ecosystems. This capability can support critical management activities, from the early detection of harmful algal blooms to the assessment of long-term trends in water clarity, which were previously impractical or impossible. The ability for an environmental officer or a local researcher to generate quantitative water quality maps using freely available software and satellite data represents a profound shift in capacity, supporting national environmental reporting and progress toward global sustainability goals.

This democratization is not an accident but the result of a deliberate strategic decision: building the toolbox as a free and open-source plugin for QGIS. QGIS is the de facto standard for open-source geographic information systems, with a massive and active user base that includes countless government agencies, non-profit organizations, and academic institutions worldwide, especially in the Global South. By leveraging this existing, widespread ecosystem, the tool’s developers have ensured its immediate availability and relevance to the very communities that stand to benefit most from it. This choice acts as a force multiplier, dramatically amplifying the tool’s potential reach and impact compared to a standalone application or a proprietary system.

Furthermore, the project’s commitment to open science, manifested through its open-source license and public hosting on GitHub, positions the RS-WaterQuality Mapper not as a static product but as a living, community-driven platform. This approach is an explicit invitation for collaboration, encouraging users from around the world to contribute their expertise by reporting issues, suggesting enhancements, or developing and sharing new, region-specific algorithms. This collaborative model fosters a dynamic ecosystem where the tool can evolve in response to emerging scientific challenges and community needs. In doing so, the toolbox also serves as a reference implementation for the principles of Findable, Accessible, Interoperable, and Reusable (FAIR) science. The transparent, auditable, and reproducible workflows it generates ensure that the resulting data products and models can be more easily shared, understood, and built upon by others, thereby strengthening the scientific foundation for evidence-based policy and management decisions.

### Acknowledged limitations and future research horizons

A rigorous assessment requires distinguishing between geometric and radiometric challenges in inland water monitoring. The toolbox relies on moderate-resolution sensors (10–30 m), which introduce spectral mixing at the land-water interface. In these “mixed pixels”, the recorded signal is a linear combination of water and bright shoreline features within the sensor’s instantaneous field of view. The toolbox currently addresses this geometrically via negative buffering (mask erosion) to physically exclude the shoreline mixing zone. However, this is distinct from the adjacency effect, a radiometric phenomenon where light reflected from bright terrestrial targets scatters into the sensor’s field of view over darker water pixels, even those not geometrically mixed with land. To mitigate this, the toolbox integrates the ACOLITE software, which includes the RAdCor processor. Unlike simple masking, RAdCor specifically addresses adjacency effects by modeling the atmospheric point spread function to estimate and remove the scattering contribution from nearby land, offering a physical correction superior to geometric exclusion alone.

Furthermore, users must be aware of optical noise sources beyond the water column. The current algorithms generally assume an optically deep water column dominated by Case-2 mixtures of chlorophyll-a, suspended sediments, and CDOM. In shallow, transparent waters, bottom reflectance may confound these retrieval algorithms, introducing uncertainties where the substrate signal mimics water constituents. Similarly, the presence of submerged aquatic vegetation (macrophytes) introduces spectral noise that can be misinterpreted as algal signals. While the toolbox’s masking protocols help exclude emergent vegetation, submerged macrophytes remain a challenge requiring careful interpretation of results in littoral zones.

Finally, the choice of atmospheric correction remains a primary source of uncertainty. As highlighted by the ACIX-Aqua project, the performance of correction algorithms varies significantly by optical water type. We integrated ACOLITE specifically to avoid the pitfalls of standard land-surface reflectance products (e.g., LaSRC), which are optimized for vegetated surfaces and often fail over water due to low signal-to-noise ratios and rigid aerosol assumptions. Future updates to the toolbox aim to incorporate newer correction methods, such as the Genetic Algorithm-based Atmospheric Correction (GAAC) (Pan & Bélanger 2025), to further improve robustness in optically complex environments.

These limitations point directly toward a clear and exciting roadmap for future development, which is enabled by the tool’s foundational design. The most critical feature ensuring the long-term viability and relevance of the RS-WaterQuality Mapper is its modular and extensible architecture. This design principle means the framework is not locked into current sensors or algorithms but is “future-proofed” to adapt and grow as the field of remote sensing advances. A key priority is the integration of hyperspectral data from next-generation missions such as NASA’s Plankton, Aerosol, Cloud, ocean Ecosystem (PACE) satellite. Supporting hyperspectral data would unlock more sophisticated applications, such as differentiating between algal functional types or more accurately characterizing complex mixtures of dissolved and suspended matter. The roadmap also includes expanding support for sensors with higher temporal frequency, like Sentinel-3 OLCI or MODIS, which are ideal for monitoring large-scale dynamics in great lakes and coastal systems.

To ensure the long-term maintenance of the software, RS-WaterQuality Mapper is released as an open-source project on GitHub. We handle the complexity of dependencies (e.g., ACOLITE, Graphviz) through loose coupling—requiring users to simply point to external executables—rather than rigid embedding. This reduces breaking changes when external tools update. For user support, we provide a comprehensive User Manual with step-by-step tutorials. We rely on the GitHub “Issues” tracking system to facilitate community-driven bug reporting and feature requests, ensuring the tool evolves through collaborative contribution rather than relying solely on the original authors.

## Conclusion

In this work, we have presented the RS-WaterQuality Mapper, an open-source QGIS plugin that successfully bridges the critical gap between advanced remote sensing science and its practical application in water resource management. The toolbox moves beyond a collection of disparate scripts to offer a cohesive, end-to-end framework that addresses long-standing barriers of workflow fragmentation, methodological complexity, and limited accessibility. Its efficacy and generalizability were rigorously validated across a diverse suite of challenging aquatic environments, from a bloom-prone temperate reservoir to a productive tropical lake and a dynamic river system, consistently demonstrating robust performance.

The core contributions of this work are twofold. Methodologically, it introduces the innovative spectral-space partitioned ensemble (SPE) model, which enhances predictive accuracy in optically complex waters, and it provides “methodological scaffolding” that makes expert-level processes like aqua-focused atmospheric correction accessible to non-specialists. Strategically, it democratizes access to satellite-based analytics by embedding these powerful tools within the globally ubiquitous QGIS platform, a choice that acts as a force multiplier for its adoption and impact, particularly in data-sparse regions.

The RS-WaterQuality Mapper is presented not as a static product but as a living, community-driven platform designed for collaborative growth. Its open-source nature and modular, “future-proofed” architecture ensure it can evolve to incorporate next-generation sensors and algorithms, fostering a global community of practice dedicated to advancing water science. By translating complex remote sensing capabilities into an accessible, transparent, and scientifically rigorous tool, this work empowers a new generation of researchers, managers, and citizen scientists to monitor and protect our planet’s most vital aquatic ecosystems more effectively.

## Data Availability

The data used for this study are openly available in Mendeley Data at the following URL [http://doi.org/10.17632/ptw96mkcpt.1](http:/doi.org/10.17632/ptw96mkcpt.1) (Su 2025).

## Code availability

The RS-WaterQuality Mapper (version 1.1) is openly available on GitHub at https://github.com/pkushb/RS-WaterQuality-Mapper along with documentation and example data. It is also from QGIS Plugin Repository at https://plugins.qgis.org/plugins/RSWaterQualityMapper/ for easy installation.
